# Sternomanubrial reduction with plating for fully displaced sternal fracture: A systematic review

**DOI:** 10.1002/ccr3.7740

**Published:** 2023-08-31

**Authors:** Sajjaad H. Samat, Krishna Yelleswarapu, Kirill Zakharov

**Affiliations:** ^1^ Department of Surgery Michigan State University Lansing Michigan USA; ^2^ Michigan State University College of Osteopathic Medicine East Lansing Michigan USA; ^3^ Thoracic and Cardiac Surgery, Sparrow Hospital Lansing Michigan USA

**Keywords:** cardiothoracic surgery, manubrium, sternal fracture, sternal plating, sternum, trauma

## Abstract

Sternal fractures are commonly due to blunt force trauma and reduction is an invasive surgical procedure typically indicated for refractory pain sternal instability. There were various modalities used for treatment and fixation of the sternal fractures. Sternal displacement fractures are traumatic injuries that may require surgical correction.

## Graphical abstract

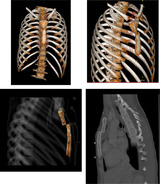

## 1 INTRODUCTION

Sternal fractures are commonly due to blunt force trauma. They occur in approximately 3%–8% of all blunt trauma resulting in complete dislocation.^1^ Sternomanubrial reduction is an invasive surgical procedure typically indicated for refractory pain, respiratory distress, nonunion of the joint, and sternal instability.^2^ Optimal therapy is debated within the field. In this study, a case report of sternomanubrial reduction with SternaLock Fixation System is presented after a total transverse sternal displacement at the manubrium. A systemic review of the published literature on sternal plating was conducted and data were generated for analysis.

## 2 METHODS

Retrospective literature search was conducted through Pubmed, SCOPUS, and Cochrane databases.

## 3 SEARCH STRATEGY

The review was performed according to the Preferred Reporting Items for Systemic Review and Meta‐Analysis (PRISMA) guidelines. Two of the authors independently performed a literature search in the PUBMED (NLM NIH) databases using the keywords “Displaced Sternal Fracture Fixation” and “Plating for Displaced Sternal Fractures”. Databases were searched from inception until July 1, 2022. The search was limited to human case reports and case series with no limitations to the date of publication, language, and text availability. The references from the articles obtained were reviewed and additional relevant papers were hand searched and reviewed.

## 4 SELECTION CRITERIA

All case reports and case series involving patients with sternal fixation after sternal displacement fractures were included in the review (Table 1).

**TABLE 1:** Reported cases of sternal plating.

Authors	Year	Average age	Sample size	Sex	Sx	Location	Cause of injury	Workup	Tx
Nijs and Broos PL^3^	2005	9	1	M	Chest pain, dyspnea, dysphagia	Sternomanubrial joint	Gymnastics	Radiograph, CT scan	L‐shaped plates
Al‐Qudah^4^	2006	28	4	M	Painful tenderness, hemothorax, rib fractures, pneumothorax	Sternal body; manubrium	MVA	Radiograph, CT scan	T‐shaped stainless steel plating
Gallo et al.^5^	2006	50	1	M	Chest pain radiating to neck and right shoulder	Sternomanubrial junction	Parachute landing	CT scan	Titanium plate fixation
Reuling et al.^6^	2015	53	1	F	Multiple rib, lumbar and thoracic vertebrae fractures, multiple myeloma	Sternum	Multiple myeloma	Radiograph, CT scan, [99 m]Tc‐MDP bone scintigraphy	Locking compression plate
Miller et al.^7^	2017	52	1	M	Fractured manubrium, rib fractures, fractured scapulae, pneumothoraces, lung contusions	Manubrium	Soil bank collapse	CT scan	Distal clavicle locking J plates
Gao et al.^8^	2018	49.2	6	5 M, 1 F	Chest pain	Sternum	Sternal tumor	Radiograph	Titanium sternal fixation system
Madjarov et al.^9^	2018	25; 29	2	M	Manubrialsternal dislocation, transverse sternal fracture	Manubrium and sternum	MVA; surfing	CT scan, radiograph	Longitudinal rigid polymer fixation
Sarkeshik et al.^10^	2019	35	1	F	Chest pain, vertebral fractures	Manubriosternal joint	MVA	Cardiac enzymes, EKG, transthoracic echocardiography, CT scan	SternaLock fixation plates
El‐Akkawi et al.^11^	2019	48	1	M	Flail chest, costal fractures, sternum and manubrium fractures, bilateral lung contusions, left tibial and calcaneus fracture	Manubrium and sternum	MVA	CT scan	MatrixRIB Fixation System
Salehi et al.^12^	2019	31	1	M	Facial bruises, mild right‐sided hemothorax	Sternum	Bicycle accident	Radiograph, CT scan	Sternal reduction with two steel parallel seven‐hole plates
Fuke et al.^13^	2021	40; 70	2	M	Flail chest; fulminant myocarditis	Sternum	MVA	CT scan	Modified Robicsek technique combined with SternaLock

Abbreviations: F, female; M, male; Sx, symptom; Tx, treatment.

## 5 DATA EXTRACTION

All selected articles were reviewed, and the following data were retrieved: age, sex, presenting symptoms, cause of injury, hospital workup, and surgical treatment modality. We also extracted authors' names and year of publication of the papers.

## 6 STATISTICAL ANALYSIS

Descriptive statistics were used to present the demographic and clinical features of the pooled data from all the selected studies. Continuous variables were presented as mean with standard deviation while categorical variables were presented as proportions. Statistical analysis was performed using GraphPad version 8.0 software.

## 7 CASE PRESENTATION

A 24‐year‐old female was admitted to the emergency department (ED) after a motor vehicle accident with a severely displaced sternomanubrial joint with persistent pain despite maximal pain control. Patient had an initial Glasgow Coma Scale (GCS) score of 15 upon presentation to the ED. The initial diagnostic workup with chest radiographs and computerized tomographic (CT) scans of the chest, abdomen, and pelvis showed small right‐sided pneumothorax which was treated conservatively without a chest tube, bilateral lung parenchymal hemorrhages, and an isolated fully displaced sternomanubrial joint without rib fractures (Figure 2). Trauma workup resulted in elevated cardiac enzymes with normal 12‐lead electrocardiogram, and transthoracic echocardiography. The patient did not have any rib fractures. On Day 2 of admission, the patient was transferred to the intensive care unit (ICU) for respiratory support after experiencing difficulty with breathing secondary to pain.

Due to refractory pain and persistent requirement of respiratory support the patient was taken to the operating room for a sternomanubrial joint reduction with fixation and plating. A 6 cm incision was made over the sternomanubrial joint at the location of the dislocation. The dissection was carried down onto the sternum and the pectoralis muscle was elevated off the sternum using a bone elevator. Penetrating towel clips were applied to the manubrium and elevated to manually reduce the dislocation. The sternum was secured using two 12‐hole SternaLock straight fixation plates which were applied longitudinally bridging the sternomanubrial junction and secured with 12, 14, and 15 mm screws (Figure 3). The incision was then closed in multiple layers with a muscle layer placed over the instrumentation. Post‐procedure the patient required decreasing narcotics and ultimately was discharged home on room air.

## 8 SYSTEMIC REVIEW

**FIGURE 1:**
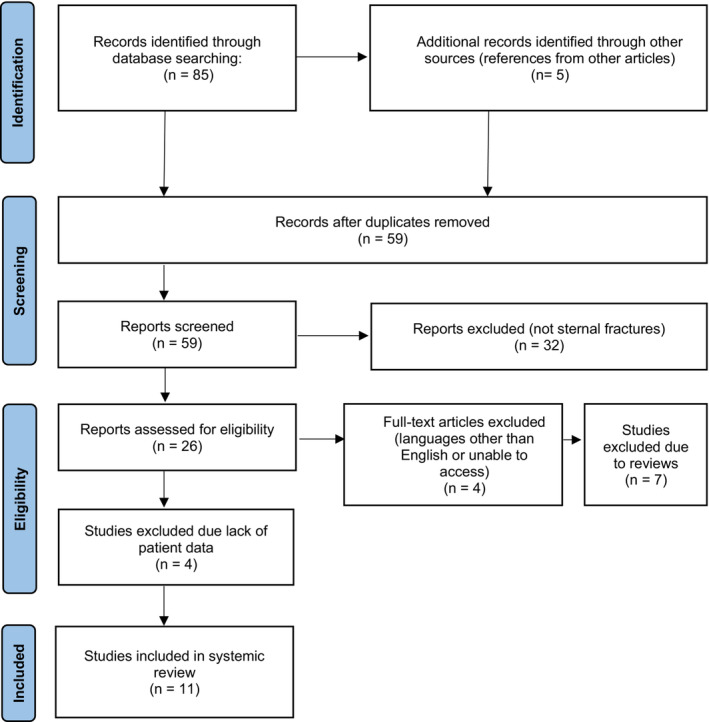
PRISMA flow diagram showing search algorithm used for systemic review.

Our literature search began with searching for key phrases “Plating for Displaced sternal fractures” and “Displaced Sternal fracture fixation” on the pubmed database. No articles were found on the Cochrane library. Of the 88 total articles, 15 met our selection criteria. Further decision was made to exclude four articles that had large sample sizes where pertinent patient characteristics were not transparent. The final number of articles included in our systemic review was 11. The PRISMA flowchart in Figure 1 summarizes the selection process.

**FIGURE 2:**
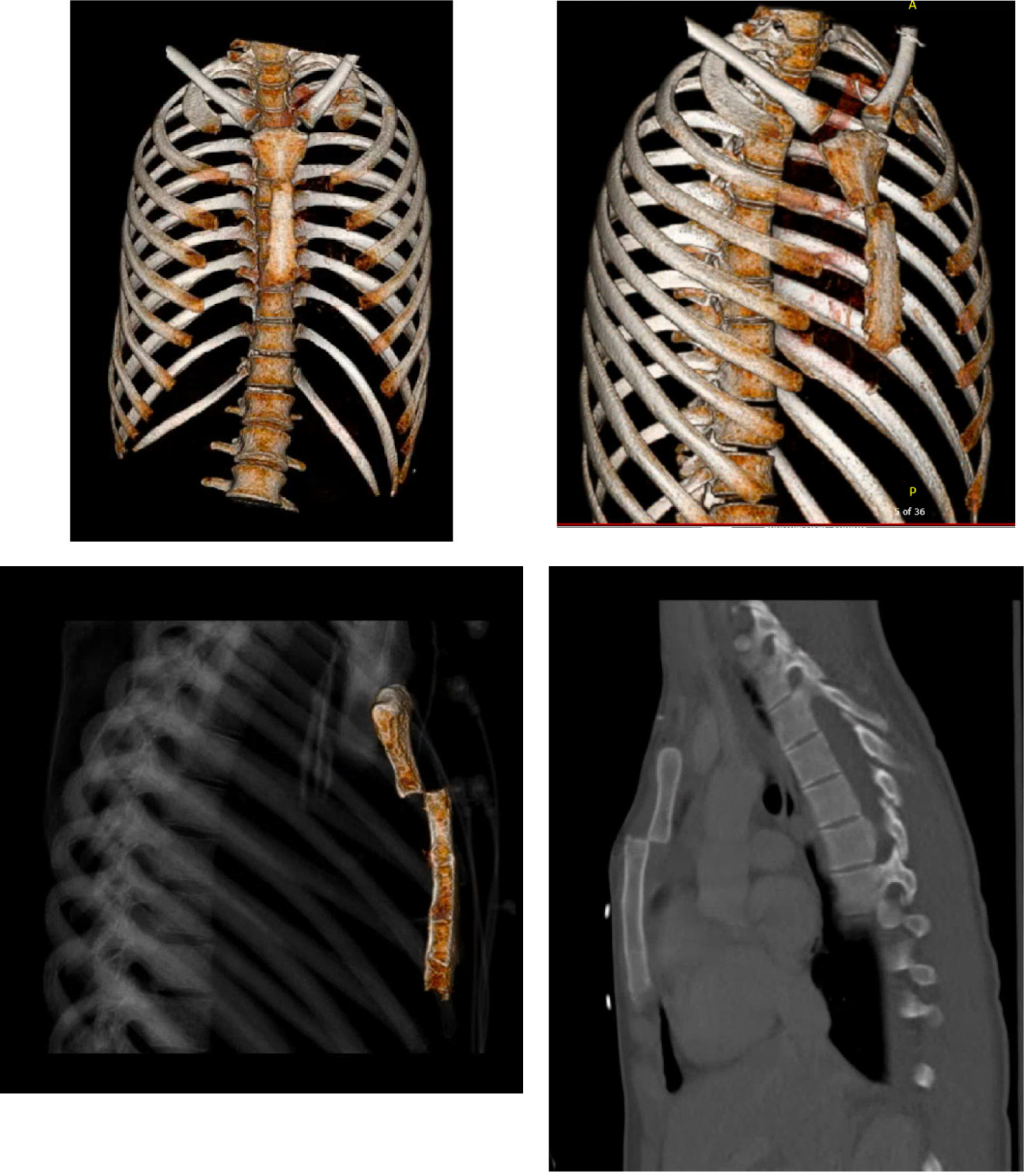
CT 3‐D reconstruction of fully displaced sternomanubrial fracture from blunt chest trauma.

**FIGURE 3:**
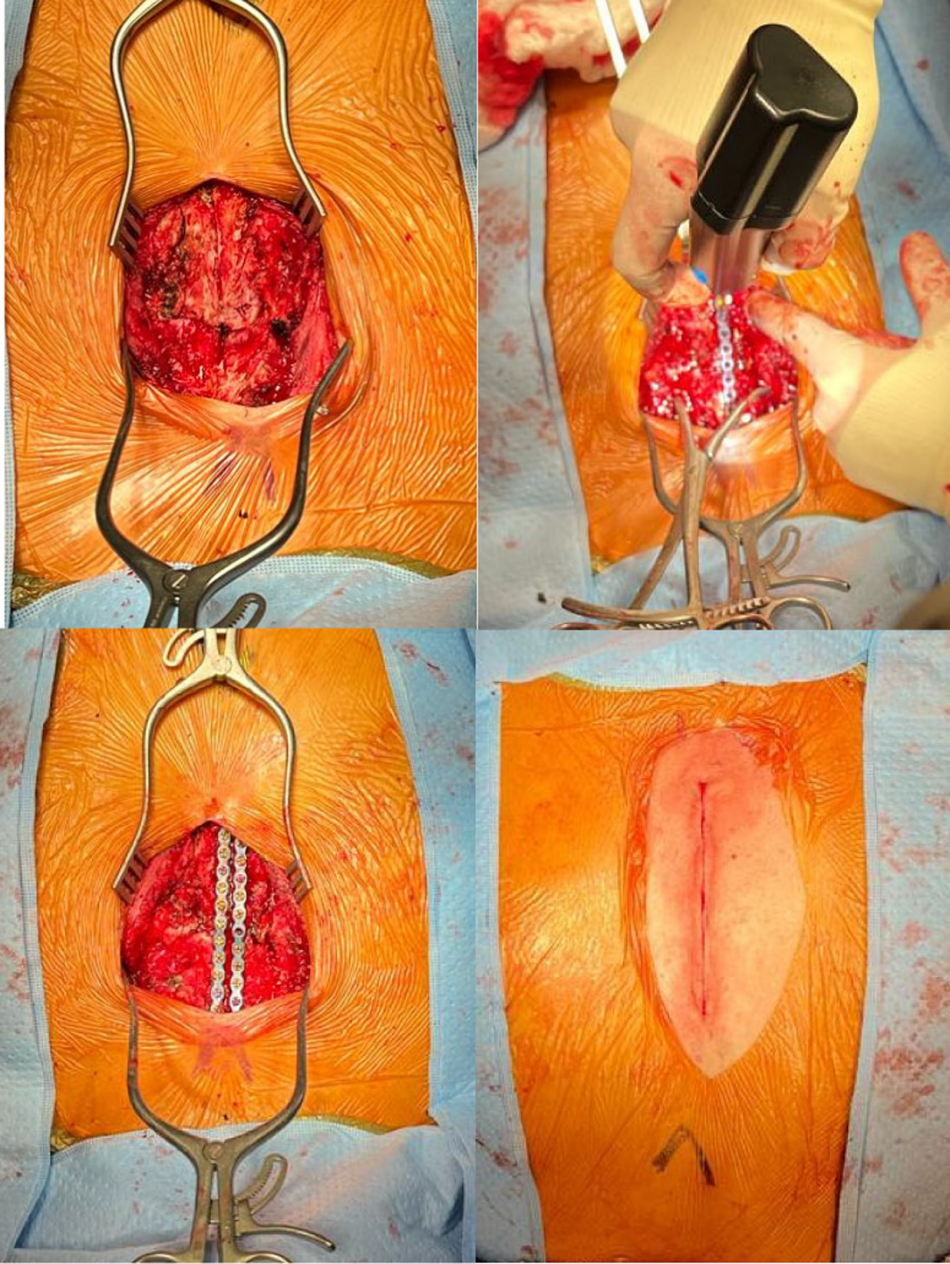
Intraoperative images with reduction of sternomanubrial joint with plating.

The systemic review articles were selected for sternal fractures; therefore, 100% of individuals had injury located at the sternum. The age of the patients at the time of diagnosis ranged from 9 years to 70 years with a mean age of 40.5 years and a median age of 40 years. Most of the cases reported were diagnosed in individuals less than 50 years of age (15 out of 21, 71.4%). Most cases were reported in male gender which constituted of 85.7% the cases (Table 2).

**TABLE 2:** Patient characteristics, symptoms, and treatments.

Characteristic	Number of patients (%)
Age	
<50 years	15 (71.4)
≥50 years	6 (28.6)
Mean age	40.5 ± 15.55
Median age	40
Gender	
Male	18 (85.7)
Female	3 (14.3)
Symptoms	
Chest pain	21 (100)
Hematoma	1 (4.8)
Rib fractures	3 (14.3)
Pneumothorax	2 (9.5)
Hemothorax	1 (4.8)
Lung contusion	2 (9.5)
Dyspnea	2 (9.5)
Flail chest	1 (4.8)
Vertebral fractures	1 (4.8)
Dysphagia	1 (4.8)
Mechanism of injury	
Trauma (accident)	13 (61.9)
Sternal tumors	6 (28.6)
Multiple myeloma	1 (4.8)
Gymnastics	1 (4.8)
Work up	
Computed tomography	15 (71.4)
Plain radiograph	9 (42.9)
Bone scintigraphy	1 (4.8)
Echocardiography	1 (4.8)
EKG	1 (4.8)
Cardiac enzymes	1 (4.8)
Treatment	
Stainless steel plating	5 (23.8)
Titanium plate fixation	7 (33.3)
Distal Clavicle J plates	1 (4.8)
Locking compression plate	1 (4.8)
Longitudinal rigid polymer fixation	2 (9.5)
MatrixRIB Fixation System	1 (4.8)
Modified Robicsek wire fixation and SternaLock	2 (9.5)
L‐shaped plates	1 (4.8)
SternaLock fixation plates	1 (4.8)

The most common symptom presented was chest pain (100%). Rib fractures presented in three patients (14.3%). Lung contusions, pneumothorax, and dyspnea were observed in two patients (9.5%) each. Hematoma, hemothorax, flail chest, vertebral fractures, and dysphagia were seen in 1 patient (4.8%) each (Table 2).

The most common cause of injury was trauma (61.9%). Trauma included motor vehicle accidents (MVA), surfing accident, chest compression injury, and collapse. Sternal tumors were the cause of injury in six patients (28.6%). Multiple myeloma and gymnastics were the cause of injury in one patient (4.8%) each.

Workup at the hospital predominantly comprised of imaging. Specifically, computer tomography scan (CT) comprised of 71.4% of the imaging modality with plain radiographs comprising of 42.9%. CT and radiographs were used simultaneously in multiple patients, and we did not distinguish it here by patient. Bone scintigraphy, EKG, and cardiac enzymes were used in one patient (4.8%).

There were various modalities used for treatment and fixation of the sternal fractures. The most common treatment was titanium plate fixation (33.3%). Stainless steel plating was the second most common used in five patients (23.8%). Longitudinal rigid polymer fixation and Modified Robicsek wire fixation & SternaLock was used in two patients (9.5%) each. Distal Clavicle J plates, Longitudinal compression plates, MatrixRIB Fixation System, SternaLock plates, and L‐shaped plates were used in one patient (4.8%) each.

## 9 DISCUSSION

Nonunion sternal dislocations can occur anywhere along the sternum. These dislocations are relatively uncommon due to need of a high velocity force. Due to the nature of injury, blunt force trauma is the most common cause of sternal dislocations. Whereas sternal fractures are benign in nature and require symptomatic therapy, sternal dislocations are almost always treated with surgery.^14^ Patients require reunion procedures that provide support and stability for the dislocated sternum to realign and heal.

The most common symptoms associated with sternal dislocations are chest pain, which is exasperated by the type of blunt trauma.^1^ Depending on the severity of the trauma, rib fractures, hemothorax, and pneumothorax can contribute to the chest pain and further complicate the patient's respiratory status. It therefore becomes imperative to act quickly and stabilize the patient.

Multiple protocols have been proposed for sternal reduction and fixation each with its advantages and disadvantages. Plating has been considered a superior choice of treatment requiring minimal dissection of the soft tissue, bringing about a closer approximation of bone and being more biomechanical compared to other reduction techniques.^15^ However, plating has also not been the preferred treatment by many cardiothoracic surgeons possibly due to their prior training or comfort with the technique.^15^ Often, plating creates a dilemma where it limits the patient's respiratory drive due to pain on the anterior chest upon inspiration.^16^

This study is limited by the nature of the previous studies used in this review. The articles used were case reports and case series that were searched on databases using the phrase “Displaced Sternal Fracture Fixation” and “Plating for Displaced Sternal Fractures”. These are level IV evidence according to the Oxford's levels of evidence. Many case reports had been excluded because of incomplete data and language other than English. Despite these challenges, this study has generated significant and relevant data about sternal dislocations and fractures with the goal of facilitating better understanding of surgical management.

## 10 CONCLUSION

Sternal displacement and fractures are traumatic injuries that may require surgical correction and necessitate an expedited hospital care of plan for patient survival and recovery. To date, there are only a few protocols for the usage of titanium fixation systems. We reckon that treatment consisting of a standardized fixation system will allow for future studies to evaluate for hospital stay, postoperative symptoms, and total recovery time.

## AUTHOR CONTRIBUTIONS

**Sajjaad H Samat:** Conceptualization; data curation; formal analysis; investigation; methodology; project administration; supervision; visualization; writing – original draft; writing – review and editing. **Krishna Yelleswarapu:** Conceptualization; data curation; formal analysis; investigation; methodology; resources; visualization; writing – original draft. **Kirill Zakharov:** Conceptualization; data curation; supervision; validation.

## FUNDING INFORMATION

This research did not receive any specific grant from funding agencies in the public, commercial, or not‐for‐profit sectors.

## CONFLICT OF INTEREST STATEMENT

The authors report no proprietary or commercial interest in any product mentioned or concept discussed in this article.

## ETHICS STATEMENT

This case report is not reviewed by the IRB as there are no identifying patient factors.

## CONSENT

Written informed consent was obtained from the patient to publish this report in accordance with the journal's patient consent policy.

## Data Availability

The data that support the findings of this study are openly available at https://doi.org/10.22541/au.166741027.74813137/v1.
